# A profile of immune response to herpesvirus is associated with radiographic joint damage in rheumatoid arthritis

**DOI:** 10.1186/ar3706

**Published:** 2012-01-31

**Authors:** John M Davis, Keith L Knutson, John A Skinner, Michael A Strausbauch, Cynthia S Crowson, Terry M Therneau, Peter J Wettstein, Eric L Matteson, Sherine E Gabriel

**Affiliations:** 1 Division of Rheumatology, Department of Medicine, College of Medicine, Mayo Clinic; 200 First Street SW, Rochester, MN 55905, USA; 2 Department of Immunology, College of Medicine, Mayo Clinic; 200 First Street SW, Rochester, MN 55905, USA; 3 Department of Radiology, College of Medicine, Mayo Clinic; 200 First Street SW, Rochester, MN 55905, USA; 4 Department of Surgical Research, College of Medicine, Mayo Clinic; 200 First Street SW, Rochester, MN 55905, USA; 5 Division of Biomedical Statistics and Informatics, Department of Health Sciences Research, College of Medicine, Mayo Clinic; 200 First Street SW, Rochester, MN 55905, USA; 6 Division of Epidemiology, Department of Health Sciences Research, College of Medicine, Mayo Clinic; 200 First Street SW, Rochester, MN 55905, USA

**Keywords:** rheumatoid arthritis, RA, immune responses, cytokines, T cells, radiographic joint damage, cytomegalovirus, Epstein-Barr virus

## Abstract

**Introduction:**

Progression of joint damage despite appropriate therapy remains a significant problem for patients with rheumatoid arthritis (RA). This study was undertaken to identify profiles of immune response that correlate with radiographic joint damage as a first step toward the discovery of new pathogenic mechanisms of joint destruction in RA.

**Methods:**

The study included 58 patients with RA and 15 healthy controls. The profiles of cytokine release from peripheral blood mononuclear cells (PBMC) in response to stimulation for 48 hours with one of six stimuli, or in media alone, were measured. Immune response profiles identified for each stimulus were correlated with radiographic joint damage as defined by the Sharp-van der Heijde score (SHS), before and after multivariable adjustment. For profiles correlated with the SHS, the distributions of individual cytokines were evaluated in patients according to the severity of joint damage and compared to healthy controls.

**Results:**

The immune response profile for cytomegalovirus (CMV)/Epstein-Barr virus (EBV) stimulation was correlated with both the SHS total and erosion scores (r = 0.31, *P *= 0.018 and r = 0.33, *P *= 0.011, respectively). After adjusting for age, sex, disease duration, autoantibody status, CMV/EBV serological status, current disease activity, disability and treatments, the correlation of the CMV/EBV immune response and the SHS erosion score became stronger (r = 0.43, *P *< 0.003). The CMV/EBV immune response correlated with CMV IgG (r = 0.44, *P *< 0.001), but not with EBV IgG. The most important cytokines for the CMV/EBV immune response profile were IFN-γ, IL-2, IL-4, IL-5, IL-13 and IL-17A, all of which are associated with T-cell immunity. Both the summary immune response score and the individual responses of IFN-γ and IL-13 to CMV/EBV stimulation were associated with greater joint damage.

**Conclusions:**

A profile of immune response to purified CMV/EBV lysates is associated with radiographic joint damage. The correlation of this immune response to CMV serology implies possible involvement of latent CMV infection. Therefore, the findings suggest that the immune response to latent CMV infection could play a fundamental role in the progression of inflammation and structural joint damage in patients with RA.

## Introduction

Rheumatoid arthritis (RA) is a systemic autoimmune disease that causes chronic, persistent joint inflammation, leading to irreversible structural damage. The etiology remains unclear, but the prevailing disease model is that multiple genes, especially HLA-DRB1 alleles, interact with environmental risk factors, including tobacco smoking, culminating in an adaptive immune response to citrullinated autoantigens that perpetuates joint inflammation [1]. T lymphocytes are key "conductors" of the inflammatory process, providing help to B cells and leading to the production of autoantibodies and the recruitment/activation of other immune effectors [2,3]. Ultimately, the activation of plasma cells, macrophages, fibroblasts, chondrocytes and osteoclasts, and downstream production of inflammatory cytokines (that is, TNF, IL-1, and IL-6), reactive oxygen species, matrix metalloproteinases and other toxic molecules, mediates destruction of articular cartilage and bone [4].

Advances in therapeutics have dramatically improved the outlook for joint structure and function in patients with RA. Current treatment strategies retard joint destruction in the majority. Nonetheless, between 5 to 30% of patients, depending on the treatment strategy used, still experience rapid progression of joint damage [5,6]. Models describing the probability of rapid radiographic progression have limited accuracy and reliability and do not fully account for joint damage [7-9]. More patients suffer slow structural deterioration over time, which can occur despite intensive treatment [10-12]. The most likely explanation is that inflammation can persist despite treatment, even when the disease appears to be in clinical remission [13-16]. Therefore, there is an urgent need to identify new pathogenic mechanisms of persistent, treatment-refractory inflammation, in hopes of developing novel targeted therapies that prevent structural deterioration over time.

We have devised an approach to discover immunological correlates of disease phenotypes based on profiles of *ex vivo *cytokine production from peripheral blood mononuclear cells (PBMC) in response to a number of non-specific stimuli. This approach was based on the concept that we could sample a broad array of activation pathways and, thereby, develop a general profile of immune responsiveness rather than define the responsiveness of specific innate or adaptive immune pathways. Our previous studies demonstrated the success of this approach by characterizing differences in the profiles of immune response between patients with early versus late RA and healthy controls, and also between patients with and without myocardial dysfunction [17,18]. The results informed our hypothesis that the broad responsiveness of one or more immune pathways is associated with the potential of the inflammatory process to mediate joint destruction. The objective of this study was to identify a profile of immune response based on *ex vivo *cytokine production that is associated with the severity of radiographic joint damage in patients with RA, after accounting for known predictors of severe disease.

## Materials and methods

### Study design and participants

We conducted a cross-sectional correlative study in the outpatient practice of the Division of Rheumatology at Mayo Clinic Rochester, MN, USA. A sample of patients referred by the 16 clinicians in the Division during the period of May 2008 through November 2009 was recruited. Adult patients of ≥ 18 years of age with a diagnosis of RA were eligible to participate if they met the following inclusion criteria: fulfillment of the 1987 American College of Rheumatology classification criteria for RA and seropositive status for rheumatoid factor (RF), anti-citrullinated protein antibodies (ACPA), or both. Patients with any of the following were excluded: clinically apparent acute infections, known chronic infections (that is, hepatitis C), recent malignancies in the past two years (excluding non-melanoma skin cancers), radiation or chemotherapy in the past two years, history of advanced chronic kidney disease or kidney transplantation, history of chronic liver disease, recent major surgery, or use of prednisone for indications other than RA. For controls, we included data on healthy blood donors from our initial study, which used identical methods as the present one [17]. The Mayo Foundation institutional review board approved this study, which was conducted according to the principles of the Declaration of Helsinki. All patients provided written informed consent.

### Data collection

One consultant rheumatologist (JMD) evaluated tender and swollen joints using modified 28-joint counts [19] and the physician global assessment of disease activity using a 0 to 100 mm visual analog scale (VAS). Patient reported outcomes included levels of pain and fatigue (0 to 100 mm VAS), duration of morning stiffness (minutes), the Health Assessment Questionnaire (HAQ) disability index [20,21], and the Medical Outcomes Study Short-Form 36 (SF-36) [22,23]. Disease activity was defined by the Disease Activity Score in 28 joints, four-variable version, using C-reactive protein (DAS28) [19]. Data were collected on patient demographics, disease duration, RF and ACPA status, and current use of disease-modifying antirheumatic drugs (DMARDs), including biologic response modifiers. C-reactive protein (CRP) was measured by turbidometric assay (Roche, Indianapolis, IN, USA). Enzyme-linked immunosorbent assays were done to assess past CMV infection using the VIDAS^® ^CMV IgG (bioMerieux, Inc., Durham, NC, USA), and multiplexed immunoassays were done to assess past EBV infection using the BioPlex™ 2200 System EBV IgG and EBV IgM (Bio-Rad Laboratories, Hercules, CA, USA).

### Radiographic scoring

Radiographs, including single views of both hands and feet, were obtained using a standardized research protocol. A board-certified musculoskeletal radiologist (JAS) interpreted all radiographs blinded to clinical and experimental information, according to the modified Sharp-van der Heijde score (SHS) system [24,25]. Separate evaluations were done as planned *a priori *for the total SHS as well as erosion and joint space narrowing (JSN) scores.

### Immune response assays

Our approach to quantify the broad responsiveness of *ex vivo *cytokine production was previously described [17]. Functional peripheral blood mononuclear cells (PBMCs) were procured by Ficoll density gradient centrifugation. Within one to two hours, 4 × 10^5 ^PBMCs were cultured in 200 μL of medium (RPMI-1640 + 10% fetal bovine serum + 1× penicillin, streptomycin, and glutamine) in the presence of one of a panel of six stimuli, or in medium alone, in a 96-well culture plate. The rationale for the use of each stimulus was detailed in our previous publication [17]. The stimulation panel included immobilized anti-CD3 and anti-CD28 monoclonal antibodies (anti-CD3/anti-CD28; Dynabeads Human T-Activator, Invitrogen, Grand Island, NY, USA); bacterial CpG oligonucleotides (CpG); combined lysates of purified CMV and EBV, containing both viral peptides and DNA (CMV/EBV; Advanced Biotechnologies, Columbia, MD, USA); PMA with ionomycin (PMA/ionomycin; Sigma, St. Louis, MO, USA); phytohemagglutinin (PHA; Sigma); and staphylococcus enterotoxins A and B (SEA and SEB; Toxin Technology, Sarasota, FL, USA). The final concentrations of each stimulant were as follows: anti-CD3/anti-CD28, 0.5 × 10^6 ^beads per culture well (1:1 ratio of beads to PBMCs per manufacturer's instructions); PHA, 5 μg/mL; *Staphylococcus *enterotoxin A, 10 ng/mL, with *Staphylococcus *enterotoxin B, 10 ng/mL; CMV, 1 μg/mL, with EBV, 1 μg/mL; CpG, 10 μg/mL; PMA, 1 μg/mL, with ionomycin, 700 ng/mL. The PBMCs were incubated at 37°C in 5% CO_2 _for 48 hours; the supernatants were then harvested, transferred to a storage plate, and frozen at -80°C for subsequent analysis.

Multiplexed cytokine analysis was performed to measure cytokine release from the PBMCs into the culture supernatants in response to stimulation using the MSD^® ^96-Well MULTI-SPOT^® ^Human Cytokine Assays tissue culture kit (Meso Scale Discovery (MSD), Gaithersburg, MD, USA). The cytokine panel included IL-1β, IL-2, IL-4, IL-5, IL-6, IL-7, IL-8 (CXCL8), IL-10, IL-12, IL-13, IL-17A, IFN-γ, TNF-α, MCP-1 (CCL2), MIP-1β (CCL4), G-CSF, and GM-CSF. Using the Sector 2400 instrument (MSD) and manufacturer-supplied reagents, the cytokine concentrations were determined based on a standard curve generated for each plate. The results were analyzed using the Discovery Workbench Software v2.0 (MSD). Previous studies have demonstrated acceptable reproducibility relative to the high degree of informative, biological variation [17].

### Statistical analysis

The patient characteristics were summarized as median (interquartile range (IQR)) or number (%). Mixed effects models were used to normalize the cytokine data as previously described [17]. In brief, fixed effects were included for age, sex, cytokine-plate lot, and random effects were included for subject and individual cytokine plate used. This resulted in effective normalization of assay effects and adjustment of the cytokine data for age and sex.

The analytic approach was based on the premise that each stimulus activates a finite number of immune pathways. The foundational concept of principal components analysis (PCA) is that for a large number of independent variables, common pathways can be identified by variables that are highly correlated. We used PCA to identify immune response profiles associated with each stimulus based on the first and second principal components. To create stimulus-cytokine PCA scores for the first and second principal components of each stimulus, all cytokine concentrations were converted to Z-scores (the mean cytokine value for the patients was subtracted from each individual cytokine value, and this difference was divided by the standard deviation of the cytokine value), then the Z-scores were multiplied by the component loadings and summed to obtain a single continuous score. For ease of interpretation, these scores were rescaled to range from 0 to 100.

The study objective was to determine the associations between key immune profiles for each stimulus and radiographic joint damage. Spearman correlations were determined between each of the stimulus-cytokine PCA scores and the SHS total, erosion, and JSN scores. Partial Spearman correlations were also assessed after adjusting for age, sex, disease duration, RF status, ACPA status, CMV/EBV serological status, RA drug treatments (methotrexate, biologic response modifiers, and prednisone), the HAQ disability index, and DAS28.

For stimulus-cytokine scores significantly correlated with joint damage, individual cytokine values with component loadings > 0.5 were compared between RA patients and controls using Wilcoxon rank-sum tests. The patients were analyzed in two groups based on a dichotomous cutoff for the total SHS of 20 units. The rationale for this particular cutoff was that the progression of radiographic joint damage by 20 Sharp units corresponds to an irreversible deterioration in physical function as defined by the HAQ disability index of 0.2, approximately the minimum clinically important difference for this outcome [26]. The rescaled stimulus-cytokine PCA score was also analyzed among the groups. Statistical significance was set at the 0.05 level; all tests were two-sided.

## Results

### Subject characteristics

The study included 58 patients with RA (Table 1) and 15 controls. On average, the patients had relatively early disease, with median (IQR) disease duration of 18.8 months (11.3, 35.9). According to the selection criteria, all patients were positive for ACPA or RF; 44 (80%) were positive for ACPA, and 53 (93%) were positive for RF. Overall, the cohort had moderate disease activity, with a median (IQR) DAS28 of 4.4 (3.4, 5.4), and moderate disability, with a median (IQR) HAQ disability index of 0.5 (0.1, 1.4). Overall, 56 (97%) of the patients had erosive disease as defined by the SHS erosion score ≥ 1. The majority of patients were taking methotrexate, and nearly half were taking prednisone; only 14 were taking biologic response modifiers (12 patients were taking anti-TNF agents, and 2 patients had received rituximab). The control group included eight females and seven males with a mean (SD) age of 44.6 (16.8) years.

**Table 1:** Characteristics of 58 patients with rheumatoid arthritis

Variable	Statistic
Age, years	54.0 (47.9, 63.0)
Sex, female	34 (59%)
RA disease durations, months^†^	18.8 (11.3, 35.9)
Pain (0 to 100 mm)	30 (16, 69)
Patient global assessment (0 to 100 mm)	28.5 (12, 63)
Morning stiffness duration, minutes	30 (15, 90)
HAQ disability index (0 to 3)	0.5 (0.1, 1.4)
RF positive^‡^	53 (93%)
ACPA positive^§^	44 (80%)
C-reactive protein, mg/L	5.9 (3.0, 11.1)
CMV IgG positive	28 (48%)
EBV IgG positive	55 (95%)
DAS28	4.4 (3.4, 5.4)
Radiographic damage scores (SHS), units	
Total	18.5 (8, 37)
Erosions	11.5 (3, 30)
Joint space narrowing	4.5 (3, 12)
Medications	
Methotrexate	49 (86%)
Biologic response modifiers	14 (24%)
Corticosteroids	27 (47%)

*Values are median (interquartile range) for continuous variables or number (%) for categorical variables. ^†^The duration of disease from onset of arthritis symptoms. ^‡^Of 57 patients tested. ^§^Of 55 patients tested. ACPA, anti-citrullinated protein antibodies; CMV, cytomegalovirus; DAS28, Disease Activity Score in 28 joints, 4-variable version, using the C-reactive protein; EBV, Epstein-Barr virus; HAQ, Health Assessment Questionnaire; IgG, immunoglobulin G; RA, rheumatoid arthritis; RF, rheumatoid factor; SHS, Sharp-van der Heijde score

### Discovery of a correlation between CMV/EBV immunity and radiographic joint damage using principal components analysis

The analysis of the first and second principal components for each of the seven stimulus-cytokine combinations resulted in identification of distinct cytokine profiles; based on the analytical premise, these were considered to reflect activation of canonical immune pathways (Table 2). For CMV/EBV stimulation, the stimulus-cytokine combinations with component loadings > 0.5 were IL-13, IL-2, IFN-γ, IL-5, IL-4, and IL-17A (in diminishing importance in the PCA score). This profile clearly reflected activation of T-cell cytokine production, across canonical T-cell subsets, in response to CMV/EBV lysate. The first and second principal components explained 30.2% and 20.7%, respectively, of the variation in cytokine production from PBMC in response to CMV/EBV stimulation.

**Table 2:** Identification of immune response profiles by means of principal components analysis*

Cytokine	CMV/EBV		SEA/SEB		CPG		PMA/ionomycin		PHA		CD3/CD28		Media Alone	
	
	PC-1	PC-2	PC-1	PC-2	PC-1	PC-2	PC-1	PC-2	PC-1	PC-2	PC-1	PC-2	PC-1	PC-2
IL-17	**0.54**	0.27	**0.75**	0.40	0.31	0.11	0.38	**0.58**	0.24	0.43	**0.81**	0.23	**0.86**	
GM-CSF	0.46	**0.52**	**0.76**	0.35	0.45	0.13	**0.73**	0.36	0.34	0.20	**0.89**		**0.87**	
IL-1β	0.25	**0.74**	**0.68**	0.36	**0.95**	-0.13	**0.76**		**0.92**	0.20	0.18	**0.55**	**0.92**	
IL-2	**0.93**		0.10		-0.27		0.17	0.31	0.27	**0.73**	0.37	**0.66**	0.14	
IL-4	**0.78**		0.13	**0.77**		**0.85**		**0.86**	0.39	**0.77**	0.47	0.26		**0.90**
IL-5	**0.79**	-0.12	0.30	**0.79**		**0.77**		0.29	0.25	**0.79**	0.31	0.32	0.33	**0.71**
IL-13	**0.93**	0.12	0.39	**0.89**	-0.20	0.24	0.13	0.18	0.29	**0.87**	0.45	0.31		0.34
IL-10		-0.17		0.38		0.18		**0.81**	**0.59**	0.41	**0.56**	**0.50**	**0.56**	0.27
IL-12	0.48	-0.16			-0.26	**0.87**	-0.13	**0.96**		0.25		0.34		**0.85**
IFN-γ	**0.85**				0.36	0.28	0.13	0.25	**0.53**	**0.63**		**0.75**	0.43	0.41
MCP-1	0.48	0.27	**0.72**	0.19	**0.60**		**0.88**		**0.71**	0.29	0.15	-0.31	**0.94**	
MIP-1β	0.29	0.20	**0.57**	0.25	**0.72**		0.35	0.29	0.43	0.37	0.57	0.34	**0.67**	
IL-6	-0.21	**0.86**	**0.87**	0.23	**0.71**		**0.95**	0.15	**0.76**	0.20	0.79		**0.93**	
TNF-α	0.39	0.33	0.47	0.23	**0.91**		0.80	0.20	**0.65**	0.49	0.56	**0.67**	**0.87**	
IL-8	-0.37	**-0.81**	-0.49		**-0.77**	0.20	**-0.88**	0.23	**-0.70**	-0.23	-0.25	-0.29	-0.19	**0.56**
IL-7			0.23		-0.15		0.45		0.40	0.22		0.11		0.27
G-CSF	-0.13	**0.96**	**0.84**	0.15	**0.82**	-0.14	**0.93**	-0.17	**0.85**	0.29	0.19	-0.12	**0.86**	-0.13

*Principal components analysis was used to identify profiles of ex vivo cytokine production by peripheral blood mononuclear cells in response to stimulation. Shown are the component loadings of each cytokine in the first and second principal components of each stimulus. Bold values indicate loadings > 0.5. CD3/CD28, anti-CD3/anti-CD28 monoclonal antibodies; CMV/EBV, cytomegalovirus/Epstein-Barr virus; CPG, CpG oligonucleotides; G-CSF, granulocyte colony stimulating factor; GM-CSF, granulocyte macrophage colony stimulating factor; IFN-γ, interferon gamma; IL, interleukin; MCP, monocyte chemoattractant protein; MIP, monocyte inflammatory protein; PC, principal component; PHA, phytohemagglutinin; PMA, phorbol myristate acetate; SEA/SEB, Staphylococcal enterotoxin A/Staphylococcal enterotoxin B; TNF, tumor necrosis factor

**Figure 1:**
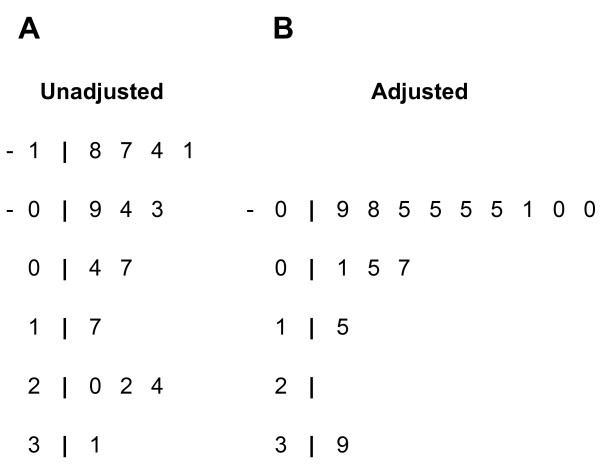
**Results of the data reduction strategy identifying immune response profiles correlated with radiographic joint damage**. Shown are stem-and-leaf schematics summarizing the hierarchy of correlation coefficients for each of the stimulus-cytokine principal component analysis scores. **(A) **Unadjusted Spearman correlation coefficients. **(B) **Partial Spearman correlation coefficients, adjusted for age, sex, disease duration, rheumatoid factor status, anti-citrullinated protein antibody status, the Health Assessment Questionnaire (HAQ) disability index, the Disease Activity Score in 28 joints (DAS28), and use of methotrexate, biologic response modifiers, and/or prednisone. The decimal point is one digit to the left of the vertical bar (for example, the bottom value in the left panel is r = 0.31). The highest unadjusted and adjusted correlation coefficients (r = 0.31 and r = 0.39, respectively) correspond to the CMV/EBV first principal component score. CMV, cytomegalovirus; EBV, Epstein Barr virus

The next step was to screen each of the immune response profiles identified using PCA for association with radiographic joint damage as defined by the SHS scores. Among all of the first and second principal components for the seven stimulation conditions, only one had a correlation coefficient of r > 0.3, a level which might be biologically meaningful: the first principal component for CMV/EBV stimulation (CMV/EBV-1) (Figure 1). Statistically significant correlations of the CMV/EBV-1 PCA score with the total SHS (r = 0.31; *P *= 0.018) and the SHS erosion score (r = 0.33; *P *= 0.011) were identified (Table 3). After adjusting for potential confounders of the relationship between viral immunity and joint damage (Table 3 and Figure 1), including markers of RA disease activity, severity and treatment status, the correlation of CMV/EBV-1 with both the SHS total and erosion scores became modestly stronger (r = 0.39; *P *= 0.006; see Table 3 for the list of covariates).

**Table 3:** Multivariable analysis of the correlation between selected immune response profiles and radiographic joint damage.*

Principal Component and Model		Sharp-van der Heijde Radiographic Score					
	
		Total		Erosions		JSN	
	
		R	*P*	r	*P*	r	*P*
CMV/EBV-1	Unadjusted	**0.31**	**0.018**	**0.33**	**0.011**	0.17	0.18
	Adjusted**^†^**	**0.39**	**0.006**	**0.39**	**0.006**	0.19	0.20
	+ CMV IgG^‡^	**0.38**	**0.009**	**0.43**	**0.003**	0.08	0.61
PMA/ionomycin-2	Unadjusted	0.24	0.07	**0.27**	**0.042**	0.15	0.27
	Adjusted**^†^**	0.01	0.93	0.09	0.53	-0.12	0.44
	+ CMV IgG^‡^	0.01	0.99	0.09	0.56	-0.14	0.36

*Correlations between stimulus-cytokine principal components scores and the Sharp-van der Heijde total, erosion, and joint space narrowing (JSN) scores were selected for presentation here based on at least one statistically significant unadjusted result. Values are Spearman correlation coefficients and associated *P*-values. **^†^**Adjusted for age, sex, disease duration, RF or ACPA positivity, HAQ disability index, DAS28, methotrexate use, biologic response modifier use, and prednisone use. ^‡^Adjusted for all covariates with the addition of CMV IgG. CMV/EBV-1, first principal component for cytomegalovirus/Epstein-Barr virus; IgG, immunoglobulin G; JSN, joint space narrowing; PMA/ionomycin-2, second principal component for phorbol myristate acetate with ionomycin

Additionally, a significant correlation of the PMA/ionomycin second principal component (PMA/ionomycin-2) with the erosion score (r = 0.27; *P *= 0.042), but not the total SHS (r = 0.24; *P *= 0.07), was identified (Table 3). After adjustment for covariates, the association between PMA/ionomycin-2 and the erosion score became non-significant (r = 0.09, *P *= 0.53). This association was confounded by the use of prednisone and disease duration, both of which correlated with PMA/ionomycin-2 (r = -0.26, *P *= 0.046 and r = 0.29, *P *= 0.025, respectively).

### Analysis of the CMV/EBV immune response profile in patients with RA and control subjects according to viral serological status

**Figure 2:**
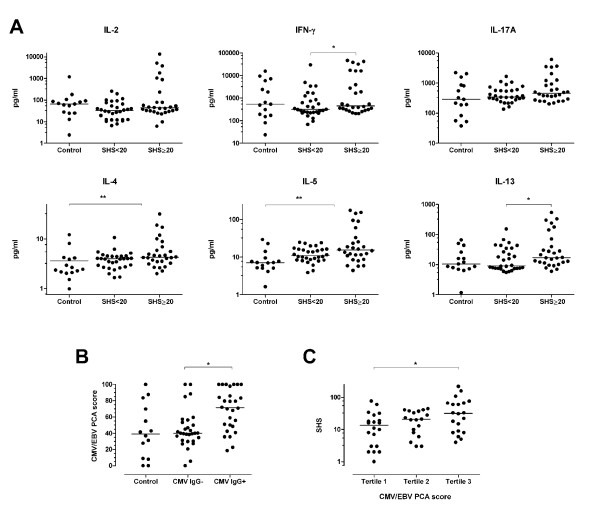
**A profile of immune response mainly to CMV correlates with radiographic joint damage in RA**. **(A) **Shown are normalized cytokine concentrations released from peripheral blood mononuclear cells into culture supernatants following 48 hours of stimulation with combined CMV/EBV lysates among healthy blood donors (Control), patients with 'low' joint damage (SHS < 20), and patients with 'high' joint damage (SHS > 20). Cytokines were selected based on the results of the principal components analysis (PCA) with factor loadings > 0.5 for the first factor. All data are adjusted for age and sex using mixed effects models. Statistical significance was tested using 2 degree of freedom tests in the mixed effects models. *P < 0.05. **P < 0.01. **(B) **Shown are the distributions of the CMV/EBV PCA score for the first principal component among healthy blood donors (Control), patients with negative CMV IgG (CMV IgG-), and patients with positive CMV IgG (CMV IgG+). *P < 0.05 for the Kruskal-Wallis test. **(C) **Shown are the distributions of radiographic joint damage defined by the total Sharp-van der Heijde score according to the tertiles of the CMV/EBV-1 PCA score. *P < 0.05 for the univariate Spearman correlation coefficient. In all panels, the horizontal lines are the medians. CMV, cytomegalovirus; EBV, Epstein-Barr virus; IFN-γ, interferon gamma; IgG, immunoglobulin G; IL, interleukin; PCA, principal components analysis; pg/ml, picograms per milliliter; RA, rheumatoid arthritis; SHS, Sharp-van der Heijde total score

The next step was to analyze the individual cytokines comprising CMV/EBV-1 among the patient groups with 'high' or 'low' joint damage and healthy controls. Significant differences in the production of T-cell cytokines were observed among the groups (Figure 2A). The RA group with high joint damage (SHS ≥ 20) had significantly increased production of IFN-γ (Th1) and IL-13 (Th2) compared to the group with low joint damage (SHS < 20). IL-2, the other Th1 cytokine studied, had a slightly lower median in the overall RA group relative to controls (statistically non-significant), yet there was a subgroup of patients with high joint damage who had markedly increased levels compared to the low-damage group or controls. The production of IL-4 and IL-5, the other Th2 cytokines, in response to CMV/EBV stimulation was significantly increased among the patients overall compared to controls. Though not statistically significant, IL-17A production also appeared to be increased overall, with both higher median and 90^th ^percentiles among the patients with RA compared to controls.

After recognizing the association of CMV/EBV immunity and joint damage, we evaluated the serological status of the patients for past CMV and EBV infection. A total of 28 (48%) had past CMV infection whereas nearly all of the patients (95%) had prior EBV infection (Table 1). Positive CMV IgG correlated significantly with joint space narrowing (r = 0.29, *P *= 0.025), but not with the total SHS (r = 0.18, *P *= 0.19) or erosion score (r = 0.10, *P *= 0.44). The CMV/EBV-1 score correlated with positive CMV IgG (r = 0.44, *P *< 0.001), but not with EBV-IgG status (r = -0.11, *P *= 0.39). This suggests that the mechanism underpinning the association between CMV/EBV immunity and joint damage is primarily specific to CMV (Figure 2B). Further adjustment for CMV-IgG status beyond RA disease characteristics (Table 3) revealed an even higher correlation of the CMV/EBV-1 PCA score with the SHS erosion score (r = 0.43; *P *= 0.003). Finally, we observed linear variation of radiographic joint damage as defined by the total SHS according to the tertile of the CMV/EBV-1 immune response score (Figure 2C).

### Correlation of CMV/EBV immune response with other clinical characteristics

To clarify the significance of the CMV/EBV immune response profile that correlated with radiographic joint damage, the next analysis considered the relationship of the CMV/EBV-1 PCA score to other clinical characteristics. The CMV/EBV-1 score was not significantly associated with the DAS28, suggesting that the correlation of CMV/EBV immunity with joint damage is not explained by clinical disease activity. Additionally, there were no statistically significant--nor biologically relevant--correlations of the CMV/EBV PCA score with age, sex, disease duration (from symptom onset), RF status, ACPA status, pain, fatigue, HAQ disability, the SF-36 physical or mental component summary scores, or treatment with methotrexate, biologic response modifiers, or prednisone (*P *> 0.2 for all; data not shown).

## Discussion

The current study represents a translational effort to discover new immune pathways underpinning structural joint damage. Our approach was to induce cytokine response profiles using a panel of stimuli designed to activate both innate and adaptive immunity, then to screen these profiles for correlation with radiographic joint damage in patients with early RA. The singular finding of this study is the association between a profile of cytokine production in response to stimulation with purified CMV/EBV and radiographic joint damage. This finding assumes significance in view of the literature supporting a potential role of latent herpesvirus infection in the pathophysiology of RA.

Both CMV and EBV are in the family of human herpes viruses, which have large double-stranded DNA genomes enclosed in a protein nucleocapsid, surrounded by a protein tegument and also an outer glycoprotein-studded lipid membrane [27,28]. Infection with these viruses is ubiquitous in people worldwide. In the United States, 50 to 60% of adults have ever been infected with CMV, whereas 90 to 95% of adults have ever been infected with EBV [27,29]. Of crucial relevance to our findings is the knowledge that herpes viruses induce a robust, primary immune response capable of controlling viral replication, yet they are still able to establish a latent infection in cell reservoirs [30,31]. CMV primarily infects monocytes and macrophages but also infects dendritic cells, epithelial cells, endothelial cells and fibroblasts [28]. EBV primarily infects B cells yet can also exist latently within nasopharyngeal epithelial cells [27]. Remarkably, 10% of the entire memory T-cell repertoire in humans, on average, is devoted to defense against latent CMV infection, underscoring the impact of this virus on the immune system [32]. Previous epidemiological studies report associations between infection with these herpes viruses (as defined by serological assays) and RA [33-38]. Indeed, latent infection with CMV and EBV is consistently detectable in the synovium [39-44], and CMV has been isolated from synovial cells of a patient with RA [45]. Studies also report the detection of viral replication in RA joints, suggesting active subclinical infection [44,46,47].

Considering herpes virus biology, our findings suggest that the immune response to latent infection with CMV or EBV may play a fundamental role in the pathophysiology of chronic inflammation and joint destruction in RA. Specifically, the key observation is that a profile of cytokine production in response to stimulation of PBMC with purified CMV and EBV lysates is associated with the severity of radiographic joint damage in patients with RA. Several pathways could give explanation for our findings. Innate immune receptors could recognize various non-self, pathogen-associated molecular patterns of the viruses [48]. Toll-like receptors (TLR) 2, 3, 7 and 9 likely have important roles in recognizing and activating responses to both CMV [49-54] and EBV [55-59]. Additionally, the protein DAI (ZBP1), and the AIM2 inflammasome are crucial sensors of viral double-stranded DNA in defense against CMV infection [48,60-63]. However, our results do not clearly suggest activation of innate immune cells by such pathways. In this case, one would expect to see production of IL-1β, IL-6, IL-8, IL-12 or TNF in response to the viruses [50,54-56], but this is not what we observed. The lack of correlation between the immune response to CpG oligonucleotide, a TLR-9 ligand and radiographic joint damage further suggests that recognition of CMV/EBV by pathogen recognition receptors and innate immune activation may not be the main explanation for our findings.

Another possible explanation for our findings is activation of cytokine production in memory T cells specific for CMV or EBV. The composition of the CMV/EBV immune profile suggests the main source of cytokine production is CD4^+ ^or CD8^+ ^T cells. These should be memory T cells, considering the high prevalence of CMV and EBV infection in our sample, in addition to the short time-period for the development of an immune response to the viruses. A key result is the significant correlation of the CMV/EBV immune response to CMV IgG^+^. The significance is that patients with RA and other immune-mediated disorders have large, clonally expanded populations of 'CD28^null^' T cells in the blood [64-67]. It happens that these CD28^null ^T cells are actually highly differentiated, effector-memory T cells, which are largely antigen-specific for CMV [64-66,68-70]. The down-regulation of the co-stimulatory molecule CD28 signifies chronic activation of the cells, which corresponds to progressive attenuation of proliferative capacity and increase in cytokine production [69,71,72]. In contrast, EBV-specific effector-memory T cells are typically CD28^+ ^[69,73]. Of further note, clonally expanded populations of CD8^+ ^T cells specific for CMV are present in RA synovial fluid [74-76]. Their frequencies are higher in synovial fluid than peripheral blood, suggesting selective enrichment of CD8^+ ^effector-memory T cells in joints [74]. Using HLA class I tetramers, CD8^+ ^T cells have been shown to react specifically with CMV pp65 epitopes [74]. Further, CMV-specific CD28^null ^T cells have a known association with the systemic complications of RA, including lung disease, vasculitis, cardiovascular disease and mortality, highlighting their potential role in the pathophysiology of RA [65,67,77,78]. In view of the evidence, CMV appears to be the more compelling of the two viruses for further study.

CMV-specific effector-memory T cells could possibly mediate inflammation in RA in many, complementary ways. They could do so in an antigen-specific manner, by targeting cells that harbor latent virus, and thus amplify the autoimmune joint disease [79]. Alternatively, CMV-specific effectors could mediate inflammation non-specifically, by migrating into inflamed joints according to chemokine gradients and becoming activated through cell-cell contact, or, by binding other cytokines in the inflammatory milieu, leading to activation of macrophage TNF production [80]. This type of 'bystander activation' of effector-memory T cells is considered a critical mechanism in RA pathophysiology [79-81]. CMV-specific effector-memory CD4^+ ^T cells are equipped to kill target cells rapidly and effectively by production of inflammatory cytokines (that is, IFN-γ or TNF) as well as perforin- and granzyme-dependent cytotoxic function [82,83]. Additionally, the expansion of CMV-specific memory T cells can overwhelm immunological niches, leading to the loss of both naïve T cells and smaller populations of memory T cells [84]. Indeed, latent CMV infection causes telomere shortening in T cells, indicative of immune senescence [85,86]. One intriguing possibility relevant to RA is that latent CMV infection is associated with increased differentiation of diverse T-cell specificities, suggesting that this could augment the function of autoreactive T cells in the joints of patients with RA [87]. Further research is necessary to elucidate the contribution of CMV-specific T-cell immunity to the progression of joint destruction in patients with RA.

This study has a number of limitations. The first is its discovery-oriented nature; our findings must be replicated in other patient populations. The study included relatively few patients currently receiving biologic therapies, limiting generalization of our findings to patients mainly on oral disease-modifying agents. Because we only collected data on current use, the results could underestimate the prevalence of ever having taken a biologic DMARD. Another limitation is the study of mixed populations of cells, precluding determination of the specific populations producing our results. We acknowledge that differences in the frequencies of cell types could contribute to our results, but in previous studies we could not explain the associations of *ex vivo *cytokine production with disease characteristics on the basis of such differences [17,18]. The use of a mixture of both CMV and EBV lysates creates uncertainties. We cannot be sure that CMV is more important then EBV in regard to RA, but overall the serological findings point to CMV as the most promising candidate for further study. The specific viral moieties that mediate the observed cytokine production are unknown. It certainly remains possible that activation of TLR pathways in innate immune cells with downstream activation of T cells could contribute to our results. Finally, this is a correlative study, so future prospective longitudinal studies are imperative to determine the causal relationship between CMV immunity and joint destruction in RA.

## Conclusion

We report the identification of a profile of *ex vivo *immune response to CMV/EBV stimulation that correlates with radiographic joint damage in patients with RA. The correlation of this immune response to CMV serology suggests the underlying drive is latent CMV infection. The nature of the response suggests the involvement of T-cell immunity. The findings assume significance in the knowledge that the magnitude of CMV-specific CD28^null ^T cells predicts severe manifestations of RA and other immune-mediated diseases. Based on our findings and the literature, we hypothesize that the immune response to latent CMV infection contributes to the propagation of inflammation and progression of structural joint damage in patients with RA. Further research is necessary to elucidate the determinants of the immune response to CMV and/or EBV that aggravate joint destruction in this disease.

## Abbreviations

ACPA: anti-citrullinated protein antibodies; anti-CD3/anti-CD28: anti-CD3 and anti-CD28 monoclonal antibodies; CMV: cytomegalovirus; CMV/EBV-1: CMV/EBV first principal component score; CpG: bacterial CpG oligonucleotides; CRP: C-reactive protein; DAS28: Disease Activity Score in 28 joints; DMARDs: disease-modifying antirheumatic drugs; EBV: Epstein-Barr virus; HAQ: Health Assessment Questionnaire; HLA-DRB1: human leukocyte antigen DRB1; IgG: immunoglobulin G; IFN-γ: interferon gamma; IL: interleukin; IQR: interquartile range; JSN: joint space narrowing; MCP-1: monocyte chemoattractant protein-1; MHV: murine gamma-herpesvirus; MIP-1β: monocyte inflammatory protein-1β; MSD: Meso Scale Discovery; PBMC: peripheral blood mononuclear cells; PCA: principal components analysis; PMA: phorbol myristate acetate; PMA/ionomycin-2: PMA/ionomycin stimulation second principal component score; RA: rheumatoid arthritis; RF: rheumatoid factor; SEA/SEB: Staphylococcal enterotoxins A and B; SF-36: Medical Outcomes Study short form 36; SHS: Sharp-van der Heijde score; TLR: Toll-like receptor; TNF: tumor necrosis factor; VAS: visual analog scale

## Competing interests

JMD, KLK and SEG are inventors of technology referenced in this article. That technology is entitled "Cytokine Response Profiling in Rheumatoid Arthritis." The Mayo Clinic has filed a provisional patent application for this technology.

## Authors' contributions

JMD, KLK and SEG designed the study. JMD recruited and clinically evaluated the patients. MSS performed the PBMC assays and multiplexed cytokine analysis. JAS interpreted and scored all joint radiographs. JMD, CSC, KLK and TMT performed the statistical analysis. All authors contributed to analysis and critical interpretation of the results as well as manuscript preparation. All authors have read and approved the final manuscript for publication.
